# Understanding Elementary Classroom Teachers’ Use of Movement Integration Resources

**DOI:** 10.3389/feduc.2020.00056

**Published:** 2020-05-28

**Authors:** Collin A. Webster, Angie Starrett, Jeffery Rehling, Brian Chen, Michael W. Beets, R. Glenn Weaver

**Affiliations:** 1 Department of Physical Education, University of South Carolina, Columbia, SC, United States,; 2 Department of Educational Studies, University of South Carolina, Columbia, SC, United States,; 3 Center for Marketing Solutions, Department of Marketing, University of South Carolina, Columbia, SC, United States,; 4 Department of Health Services Policy and Management, Columbia, SC, United States,; 5 Department of Exercise Science, University of South Carolina, Columbia, SC, United States

**Keywords:** classroom physical activity, activity breaks, comprehensive school physical activity, whole-of-school, teacher training

## Abstract

Movement integration (MI)—infusing physical activity (PA) into normal classroom time during school—is an evidence-based strategy to support public health goals and enhance educational outcomes for elementary children. However, few elementary classroom teachers in the United States appear to be using MI resources. In order to understand teachers’ MI resource use, this study’s purpose was 3-fold: (a) to examine teachers’ perceptions about MI and MI resources, (b) to identify teachers’ latent class membership based on their perceptions, and (c) to determine the extent to which teachers’ MI preferences, demographics, background, and school context predict class membership. We recruited a national sample of participants (*N* = 420) who completed an electronic survey including six sections: perceived benefits of MI, perceived barriers to using MI resources, satisfaction with MI resources, perceived importance of different MI resource characteristics, MI resource preferences, and participant demographics, background, and school context. Data analysis consisted of descriptive analyses, exploratory factor analysis, confirmatory factor analysis, latent profile analysis, and multinomial regression analysis. Participants mostly agreed about the benefits of MI and were satisfied with the MI resources they were using but had varying perceptions about the presence of barriers to using MI resources. Participants rated the integration of an MI resource with the academic curriculum, the amount of time required for teacher training, the type of training materials provided, and the timing of the training as the most important characteristics they would consider before adopting a resource. Factor analyses supported single factor solutions for perceived benefits, perceived barriers, and satisfaction, and a three-factor solution for perceived importance of different MI resource characteristics (Curriculum Integration, Training Logistics, and Feasibility). Based on these factors, we identified five latent classes of teachers. Regression results showed that desirable latent class membership depended on certain MI preferences and demographic, background, and school context characteristics. Teacher educators and interventionists should consider teachers’ MI perceptions, the nature of MI trainings, teacher characteristics, and school context in efforts to increase teachers’ use of MI resources. Further research is needed to explore the intrinsic value of MI for teachers, given MI’s education- and public health-related benefits.

## INTRODUCTION

Classroom time in elementary schools includes movement opportunities resulting from the physical environment, transitions, breaks from academic lessons, or learning activities that involve physical activity (PA; Russ et al., 2017; Stewart et al., 2019). Movement opportunities may occur inside or outside of the classroom setting and can be teacher-directed or student-initiated (Webster C. et al., 2015; Russ et al., 2017). Various terms apply to instances when PA is incorporated into classroom time, such as “classroom-based PA,” “PA breaks,” and “active lessons.” Webster C. et al. (2015) refer to the compendium of all such instances as *movement integration* (MI) to conceptualize the broad range of methods and manifestations related to evoking students’ PA engagement during normal classroom time.

In the context of public health, MI presents a unique platform for increasing children’s access to PA opportunities during school. It is common for elementary students to spend more hours at school with their classroom teachers than with any other teacher; therefore, MI has unparalleled reach as a school-based strategy for increasing children’s daily PA (Webster C. et al., 2015). Combined with physical education, recess, and before- and after-school opportunities, MI is viewed as a promising way to help children achieve the nationally recommended 60 min of PA each day (Institute of Medicine, 2013; Centers for Disease Control and Prevention, 2017). Specifically, classroom activity breaks can result in an average of 19 added minutes per day of moderate-to-vigorous PA (Bassett et al., 2013).

MI also is appealing in the context of educational reform, which has placed ever-increasing pressure on schools to produce higher levels of academic achievement since the early 1980s (Mehta, 2015). Consistently, MI positively associates with students’ on-task behavior and performance on standardized tests (Erwin et al., 2012; Watson et al., 2017). MI’s emergent link to both health-related and educational outcomes has spurred the development of numerous recommendations (e.g., how to use MI as part of a larger, schoolwide PA plan; what to consider when thinking about trying new MI approaches) and resources (e.g., specially-designed active classroom furniture, PA task cards, pre-designed academic lessons with integrated PA) aimed at helping classroom teachers capitalize on, enhance, and increase MI to maximize its impact on children’s daily PA and academic learning.

We designed the present study to understand elementary classroom teachers’ use of MI resources. The availability of resources is a commonly reported facilitator for MI, whereas a lack of resources stands as a persistent barrier (Michael et al., 2019). Helping teachers overcome perceived barriers to MI may increase the extent to which teachers capitalize on existing movement opportunities for students or try new MI methods. In a national survey, less than half of elementary schools in the U.S. reported providing PA breaks (Centers for Disease Control and Prevention, 2019), which may be indicative of a general trend in which teachers are choosing not to use certain MI resources. Understanding teachers’ use of MI resources can help to inform the work of teacher educators and interventionists who strive to promote school-based PA. Specifically, teacher educators will be better able to help teacher candidates and inservice teachers feel comfortable with and adopt MI into classroom routines by focusing on MI resources that are most appropriate to teachers’ interests and needs. Interventionists will be able to deliver MI trainings that provide teachers with resources that are best suited to particular types of teachers and school contexts.

Our aim in the present study was 3-fold: (a) to examine teachers’ perceptions about MI and MI resources, (b) to identify distinct teacher profiles based on teachers’ perceptions about MI and MI resources, and (c) to determine the extent to which teachers’ MI preferences, demographics, background, and school context characteristics predict teacher profiles. Specifically, we asked the following research questions:

1. What are teachers’ perceptions about MI and MI resources, including perceived benefits of MI, perceived barriers to using MI resources, satisfaction with MI resources, perceived importance of different MI resource characteristics with respect to resource adoption, and MI preferences?
2. What latent classes (i.e., distinct teacher profiles) exist based on teachers’ perceived benefits of MI, perceived barriers to using MI resources, satisfaction with MI resources, and perceived importance of different MI resource characteristics with respect to resource adoption?
3. To what extent do teachers’ MI preferences, demographics, background, and school context characteristics predict class membership?

## METHOD

### Participants

Participants were 420 elementary classroom teachers who self-reported using one or more MI resource. Participants’ demographic, background, and school context information is presented in Table 1.

### Instrumentation

We employed an electronic survey to assess participants’ (a) perceived benefits of MI, (b) barriers to using MI resources, (c) satisfaction with these resources, (d) perceived importance of different MI resource characteristics with respect to resource adoption, (e) MI resource preferences and (f) demographics, background, and school context.

#### Perceived Benefits of MI

Eleven items, adapted from previous research (Webster et al., 2013, 2017), assessed participants’ perceptions of the benefits of implementing MI. Items employed a 7-point Likert scale from “Strongly Disagree” to “Strongly Agree.” The stem, “As you think about movement integration as a part of your classroom routines, how strongly do you agree or disagree?” preceded all items. Example items are “Implementing movement integration enhances students’ knowledge about the importance of physical activity,” “Providing movement integration enhances my performance as a teacher,” and “Offering movement integration enhances how our school is viewed by other schools in our district.”

#### Perceived Barriers to MI Resource Use

Six items assessed participants’ perceptions of the barriers to incorporating MI into their classroom routines. Questions included barriers to MI identified in previous research (Bartholomew and Jowers, 2011; Webster et al., 2013, 2017). The stem, “On a scale of 1 to 5 with 5 being “Major barrier” and 1 being “Not a barrier at all,” how would you rate each of the following barriers as challenges to using movement integration products in your classroom routines?” preceded all items. Items included “Lack of information/knowledge about movement integration,” “Lack of formal movement integration training,” “Cost,” “Limited instructional time,” “Limited planning time,” and “Not my responsibility.”

#### Satisfaction With MI Resources

Two items assessed participants’ satisfaction with MI resources they were using. On a 4-point Likert scale from “Extremely satisfied” to “Extremely dissatisfied,” participants responded to the following items: “How satisfied are you with the current MI approach/products you are using?” and “How likely are you to recommend the product you are using to other classroom teachers?”

#### Perceived Importance of Different MI Resource Characteristics

Eight items assessed the importance of different characteristics of MI resources. Instructions stated for teachers to rank, from most to least important (1 = most important; 8 = least important), the influence that specific resource characteristics had on their choice to acquire and integrate a resource into their classroom. Ranked characteristics included the amount of time required for teacher training, the type of training materials provided (online vs. hard copy, in person vs. virtual), timing of training (before or after the school day, beginning or end of the year), integration of academic content into the curriculum (aligns with other academics, use, delivery), delivery location (classroom, lab, outdoors), length of time the activities in the resource lasted, cost of resource, and specific materials available/included with a resource.

#### MI Resource Preferences

Ten items assessed participants’ preferences with respect to MI resources. Items focused on the cost of current resource/s (free, <$50, $50–$99, $100–$199, $200–$299, $300–$399, $400–$499, over $500, Don’t know/Unsure), optimal training length (no training is required, 60, 90 min, half day, full day, multiple days), training delivery method (none, hard copy/paper, digital manual, virtual/online), training support (in person, webinar, videos, external support), training time of year (prior to the start of the school year, beginning of the school year, end of school year, following the end of the school year, mid school year, other, communications throughout the year) and time of day (before the school day starts, during the school day, after the school day). Items also focused on participants’ preferred approach to integrating movement (integrated within academic lessons/content, as a break from/transition between academics), preferred implementation method (technology-led [online video or game], teacher-led [you lead students through an activity], student-led [students lead other students through an activity], equipment-led [standing desks, desks that allow children to peddle, chairs that allow children to wobble/move]), preferred implementation location (general classroom, activity lab [room with special equipment to deliver integrated content], outdoors, gymnasium, other shared facility [cafeteria, auditorium]), and preferred implementation length (<5, 6–10, 11–15, 16–20, 21–30, over 30 min).

#### Demographic, Background, and School Context Information

Thirteen items assessed participants’ demographics, background, and school context. Demographic information included age, gender, and race/ethnicity. Background information included years of teaching experience and highest level of education achieved. School context information included the state in which the teacher lived, their geographic area (urban, suburban, rural), size of the school and district in which they taught, and type of school (public, private).

### Procedure

The Institutional Review Board at the University of South Carolina approved this study prior to data collection. We developed the survey for the purposes of this study. The literature informing item construction included product testing methods and techniques (Van Kleef et al., 2005; Graner and Mißler-Behr, 2012), previous MI survey research (Webster, 2011; Webster et al., 2013; Webster C. A. et al., 2015), published reviews of MI and school-based PA promotion (e.g., Erwin et al., 2012; Russ et al., 2015; Webster C. et al., 2015), and studies of MI resources/strategies (Kohl et al., 2001; Stewart et al., 2004; Liu et al., 2008; Donnelly et al., 2009; Bartholomew and Jowers, 2011; Donnelly and Lambourne, 2011; Webster et al., 2013; Goh et al., 2014; Webster C. A. et al., 2015). To content validate the survey items, we had two researchers who are nationally recognized for their research on MI and school-based PA promotion and an expert in the field of product testing review the items. These individuals provided three rounds of feedback on electronic copies of the survey via email and then met in person to come to consensus on item wording.

Subsequently, we drafted the full survey including item response scales and directions for participants. We defined MI in the survey using the following definition and examples (Webster C. et al., 2015):

MI is infusing physical activity, at any level of intensity, into normal classroom time, usually within the general education classroom setting. Examples of MI are: Incorporating physical activity into regularly scheduled classroom transitions (e.g., having students jog from desks to carpet or hop in place while waiting in line), taking short movement breaks during academic lessons (e.g., showing a GoNoodle video or leading a brief exercise routine), and teaching academic lessons using physically active strategies rather than traditional seatwork (e.g., having students act out stories or move into groups to demonstrate answers to division problems). Other common terms for MI include classroom physical activity, active lessons, and brain bursts.

In addition, we included a single item at the start of the survey to determine whether participants had used any MI resources in their classroom. We designed the survey so that only respondents who indicated they had used MI resources would proceed with the survey.

We pilot tested the survey with a convenience sample of five elementary classroom teachers not included in the final survey administration. These teachers completed a paper copy of the survey A research assistant noted any clarifying questions the teachers asked and any verbalized suggestions they made to enhance the clarity and readability of the survey. We then made additional revisions to finalize the survey, based on these teachers’ input.

We recruited participants through Qualtrics’ online sample recruitment tool (i.e., Qualtrics Panel). The Qualtrics Panel consisted of 12,161 teachers. We identified a total of 1094 eligible teachers (i.e., self-identified as elementary general education classroom teachers and indicated they were aware of MI) selected the final sample in order to reflect national teacher profiles of elementary school teachers. Recruiting participants in this way has been shown to produce samples and data that reflect national profiles of the United States population (Heen et al., 2014; Ibarra et al., 2018).

When selected from the Qualtrics Panel, participants received a link to complete the survey in the Qualtrics online survey platform via text or email. Participants were allowed to take as much time as necessary to complete the survey (mean completion time = 11.5, SD = 6.4 min). As approved by our Institutional Review Board and in order to maximize participant response rate, we offered a small monetary incentive to complete the survey. Consistent with past online surveys, we employed attention checks and quality screens to ensure the quality of the data (Ibarra et al., 2018).

### Data Analysis

#### Descriptive Statistics

We analyzed descriptive statistics from the survey instrument in Stata (v. 14.2, College Station TX). Specifically, we calculated response frequencies for each of the included survey items.

#### Factor Analysis

To explore and confirm factor structure for the items assessing perceived benefits, perceived barriers, satisfaction, and perceived importance of different MI resource characteristics, we used MPlus version 8 with the robust maximum likelihood (MLR) mode of estimation. MLR provides parameter estimates with standard errors robust to non-normality. We used exploratory factor analysis (EFA) to determine the possible number of factors with the Geomin rotation, an oblique rotation type, and applied multiple fit statistics to assess optimal factor structure across the different solutions. The fit indices included Akaike Information Criteria (AIC), Bayesian Information Criteria (BIC), Root Mean Square Error of Approximation (RMSEA), Comparative Fit Index (CFI), and Standardized Root Mean Square Residual (SRMR). We used these indices measure the degree to which the factor model reproduced the empirical covariance matrix. Once we found a possible optimal factor structure, we used a five-step procedure (model specification, identification, estimation, testing fit, and respecification) for the confirmatory factor analysis (CFA). The CFA used the same fit statistics as the EFA and could stop in Step 4 (testing fit) if the model fit the data well. We calculated descriptive statistics (i.e., means and standard deviations) for each of the included factors in the final factor solution.

#### Latent Profile Analysis

We used Mplus version 8.1 to perform latent profile analysis (LPA) and used ESTIMATOR=MLR, maximum likelihood parameter estimates with standard errors, which is robust to non-normality. The relative model fit indices we used were Akaike information criteria (AIC), Bayesian information criteria (BIC), the Vuong-Lo-Mendell-Rubin test (VLMR), and the Lo-Mendell-Rubin test (LMR). To determine the model with the optimal number of latent classes, we performed sequential analyses increasing the number of classes in the model by one, starting with a two-class model. We compared the fit indices of the model with the smaller number of classes to those of the model with one additional class. Better fitting models had smaller AIC and BIC values, while maintaining statistically significant VLMR and LMR values (Geiser, 2012; Lanza et al., 2013). The model with the optimal number of classes was the model with one less class than the model where the VLMR and LMR values became significant. We also considered entropy values, indicating the quality of the classification of the model, with values close to one indicating high accuracy in classification (DiStefano and Kamphaus, 2006; Collins and Lanza, 2009; DiStefano, 2012). The model with the optimal number of classes not only had the fewest number of classes (parsimony), but also took theory and logic into consideration.

#### Multinomial Logistic Regression

Prior to performing the second step of the analysis, we assigned each individual to a class from the optimal LPA solution using modal assignment. We used multinomial logistic regression in SPSS to predict teacher MI resource profiles based on the teachers’ MI resource preferences, as well demographic, background, and school context information.

## RESULTS

A total of 73 participants responded that they had not used any MI resources. Therefore, all results are for only the remaining 347 participants in the sample.

### Descriptive Data

Descriptive data, displayed as response frequencies for each survey item, are shown in Table 2. Across items assessing perceived benefits of MI, 80% of participants, on average, strongly agreed, agreed, or slightly agreed about the stated benefits. The highest level of agreement (84%) was for the item, “Implementing MI keeps my students on task better than other activities.”

On the perceived barriers questionnaire, participants’ ratings were mixed, although there was a slight lean across items toward viewing the listed factors as barriers. On average, 52% of participants rated the factors as a 4 or 5 (“major barrier”). The most pronounced divisions in participants’ perceptions were for cost (61% of participants felt cost is a barrier) and for whether taking a view that classroom teachers are not responsible for using MI presents a barrier to using MI resources (67% of participants felt such a view is not a barrier).

The majority (56%) of participants indicated they were extremely satisfied with the MI resources they were using and only 4% of participants indicated they were not satisfied. Additionally, 64% of participants indicated it was extremely likely they would recommend the MI resources they were using to other classroom teachers.

Regarding MI resource characteristics, participants rated the following characteristics as most important to informing their decision to adopt a resource (based on aggregated ratings of the top four response options on the eight-point scale): the integration of the resource with the academic curriculum (75% of participants) the amount of time required for teacher training (71% of participants), the type of training materials provided (64% of participants), and the timing of the training (62% of participants).

The majority of participants (61%) indicated the MI resources they were using cost $99 or less. Participants preferred MI trainings that were 90 min or less (67%), with the largest number of responses favoring a 60-min training (39%). Most participants (93%) preferred to receive some type of guidebook/training manual for MI resources, particularly in the form of a hardcopy (35%) or digital manual (32%). Additionally, participants’ preferred MI trainings that were in person (45%) and held prior to the start of the school year (53%) with any ongoing support during the school year provided after the school day (59%). With respect to MI implementation options, participants were evenly divided in their preference to use MI resources in ways that either integrate with academic content or provide movement as a break from academics. Participants also were partial to leading MI activities themselves (49%) or via the use of technology (31%) rather than having students lead the activities or relying on equipment (e.g., standing desks) to promote PA. Finally, participants indicated they usually used MI within their classroom space (69%) as opposed to within other school contexts, and about two-thirds of participants (67%) indicated a period of between 6 and 20 min as the duration of a typical or average occasion of MI activities.

### Factor Solutions

EFAs supported a one-factor solution for the perceived benefits, perceived barriers, and satisfaction questionnaires. Nine of the 11 items (α = 0.972) were retained from the perceived benefits questionnaire, all six items (α = 0.826) were retained from the perceived barriers questionnaire, and both items were retained from the satisfaction questionnaire. We labeled these factors “Benefits,” “Barriers,” and “Satisfaction,” respectively. The analysis supported a three-factor solution for the perceived importance of different MI resource characteristics. The single item assessing the importance of integrating the resource(s) into the curriculum loaded on its own factor, hereafter referred to as “Curriculum Integration.” The three items assessing the importance of the amount of time required for training, the type of training materials, and the timing of the training all loaded on a factor, hereafter referred to as “Training Logistics.” The three items assessing the importance of delivery location, cost, and materials available loaded on a factor, hereafter referred to as “Feasibility.”

The results of the CFAs are presented in Table 3. For Benefits, the value of RMSEA indicated excellent approximation to the data and the SRMR (0.018) demonstrated an acceptable fit (Hu and Bentler, 1999). The nine items had a reliability value of α = 0.972, and given the results of the CFA, the nine items were averaged for the LPA. For Barriers, the value of RMSEA indicated a reasonable approximation to the data, while both the SRMR and CFI demonstrated an acceptable fit. The six items had a reliability value of α = 0.826, and given the results of the CFA, the six items were averaged for the LPA. For Satisfaction, Training Logistics, and Feasibility, the final solution contained three factors. Since a latent factor requires at least two items, Curriculum Integration was excluded for the CFA analysis. To confirm the fit of the three-factor model, A CFA was run with the MLR estimator. The value of RMSEA indicated a reasonable approximation to the data, while the SRMR and CFI also demonstrated an acceptable fit. The items belonging to each factor were averaged for the LPA, and the single item for Curriculum Integration was also included.

### Teacher Movement Integration Resource Profiles

We averaged the items belonging to each factor for the LPA, and also included the single item-factor, Curriculum Integration. Table 4 provides information from consecutive LPA runs used to determine the best-fitting model. The optimal class solution was the 5-class model, which contained good entropy, significant *p*-values for both VLMR and LMR, and quantitatively well-defined classes. We attempted a 6-class solution but it never converged even when we employed maximum starts.

To substantively interpret each class, we evaluated the conditional response means and the overall sample means (see Table 5). We calculated Z-scores for each factor comprising the latent profile, and interpreted z-score values >|0.5| as quantitatively defining the class. For Class 1 (56 teachers), Benefits was nearly one standard deviation below the mean (*z* = −0.88), both Training Logistics and Feasibility were over one standard deviation below the mean (*z* = −1.28 and *z* = −1.53, respectively), and Curriculum Integration was over half a standard deviation below the mean (*z* = −0.76). Thus, teachers in this profile tended to only slightly agree with possible benefits of MI, they were likely to strongly value the importance of training for the MI resource(s), and they tended to highly value the delivery location, feasibility, and available materials of the resource(s). Also, these teachers were likely to highly value the importance of integrating the resource(s) into the curriculum.

For Class 2 (46 teachers), Barriers was nearly half a standard deviation below the mean (*z* = −0.48), Training Logistics was over one standard deviation above the mean (*z* = 1.68), and Feasibility was over half a standard deviation below the mean (*z* = −0.76). Thus, teachers in this profile on average tended to not perceive barriers as challenges to using MI resource(s), they were likely to not value the importance of training for resource use, and they tended to prioritize the delivery location, feasibility, and available materials of the resource(s). While they were similar to Class 1 teachers in that they valued delivery location, feasibility, and available materials, Class 2 teachers did so to a lesser degree than the teachers in Class 1. Class 2 was comprised of 46 teachers or 13% of the sample.

For Class 3 (71 teachers), Training Logistics was over half a standard deviation above the mean (*z* = 0.64), but Curriculum Integration was over half a standard deviation below the mean (z = −0.57). Consequently, the teachers in this profile were less likely to value the importance of training but highly likely to value the importance of integrating the resource(s) into the curriculum.

For Class 4 (50 teachers), Curriculum Integration was well over one standard deviation above the mean (z = 1.63). Thus, teachers in this profile on average did not value the importance of integrating the resource(s) into the curriculum.

For Class 5 (124 teachers), Training Logistics was over half a standard deviation below the mean (*z* = −0.57) and Feasibility was over one standard deviation above the mean (*z* = 1.03). Thus, teachers in this profile on average highly valued the importance of training for resource use, but they were less likely to prioritize the importance of delivery location, feasibility, and available materials.

### Multinomial Logistic Regression

We viewed Class 3 as the closest to average class due to the smallest z-scores between the conditional class means and the overall sample means. Therefore, we used Class 3 as the referent group in the multinomial logistic regression. In the regression, we included several teacher demographics, including gender, ethnicity, highest degree completed, and years of teaching experience. We also included several school context characteristics, including geographic area (urban, suburban, rural), district size, school type (public or private), and level of school lunch assistance for which the majority of students qualify in USDA National School Lunch Program (free, reduced, or none). Further, we included a block of teacher training variables, encompassing training length (multiple, one, or none), training delivery method, type of training support, and training time of year (during or not during school year). Finally, we considered teachers’ preferred approaches for using the MI resource(s), including when integrated, when during the day, and whether or not as a reward for good behavior, and also considered the current resource cost and participants’ preferred implementation for the MI resource(s), including the method (teacher-led, student-led, or technology-led), the location (inside or outside the classroom), and the length of use. Results from the regression analysis are presented in Table 6.

#### Comparing Class 1 to Class 3

Compared to no training, teachers receiving one training will decrease the relative likelihood of being in Class 1 (compared to Class 3) by 0.15 unit (relative probability of 13%) (95% confidence interval for *or*: 0.03–0.78). Compared to receiving one training, teachers receiving multiple training will decrease the relative likelihood of being in Class 1 (compared to Class 3) by 0.23 unit (relative probability of 19%) (95% confidence interval for *or*: 0.06–0.86). Compared to receiving a hard copy of training materials, teachers receiving no training materials will increase the relative likelihood of being in Class 1 (compared to Class 3) by 4.20 unit (relative probability of 81%) (95% confidence interval for *or*: 1.44–12.26). Compared to receiving no training materials, teachers receiving virtual/online training materials will increase the relative likelihood of being in Class 1 (compared to Class 3) by 12.98 unit (relative probability of 93%) (95% confidence interval for *or*: 2.00–84.50). Compared to during the school year, teachers receiving training outside of the school year will increase the relative likelihood of being in Class 1 (compared to Class 3) by 2.93 unit (relative probability of 75%) (95% confidence interval for *or*: 1.34–6.38). Compared to a daily opening activity, teachers who use the product(s) throughout the day will increase the relative likelihood of being in Class 1 (compared to Class 3) by 2.65 unit (relative probability of 73%) (95% confidence interval for *or*: 1.17–6.00). Compared to using the product(s) as a reward for good behavior, teachers who use the product(s) throughout the curriculum will increase the relative likelihood of being in Class 1 (compared to Class 3) by 3.19 unit (relative probability of 76%) (95% confidence interval for *or*: 1.38–7.33). Compared to using the product(s) in the classroom, teachers using the product outside the classroom will decrease the relative likelihood of being in Class 1 (compared to Class 3) by 0.30 unit (relative probability of 23%) (95% confidence interval for *or*: 0.13–0.68). Compared to having no cost, teachers using products that cost under $100 will increase the relative likelihood of being in Class 1 (compared to Class 3) by 4.88 unit (relative probability of 83%) (95% confidence interval for *or*: 1.16–20.51). Compared to costing under $100, teachers using products that cost $100 or more will increase the relative likelihood of being in Class 1 (compared to Class 3) by 7.46 unit (relative probability of 88%) (95% confidence interval for *or*: 2.03–27.38). Compared to schools in suburban locations, teachers from urban-located schools will decrease the relative likelihood of being in Class 1 (compared to Class 3) by 0.36 unit (relative probability of 26%) (95% confidence interval for *or*: 0.15–0.84). Compared to large districts, teachers at schools in medium-sized districts will decrease the relative likelihood of being in Class 1 (compared to Class 3) by 0.24 unit (relative probability of 19%) (95% confidence interval for *or*: 0.07–0.81). Compared to Caucasian teachers, minority teachers will decrease the relative likelihood of being in Class 1 (compared to Class 3) by 0.44 unit (relative probability of 31%) (95% confidence interval for *or*: 0.20–0.96). Compared to teachers with less than a Bachelor’s degree, teachers having a Bachelor’s degree will increase the relative likelihood of being in Class 1 (compared to Class 3) by 2.83 unit (relative probability of 74%) (95% confidence interval for *or*: 1.01–7.97).

#### Comparing Class 2 to Class 3

Compared to costing under $100, teachers using products that cost $100 or more will increase the relative likelihood of being in Class 2 (compared to Class 3) by 3.21 unit (relative probability of 76%) (95% confidence interval for *or*: 1.13–9.14). Compared to large districts, teachers at schools in medium-sized districts will decrease the relative likelihood of being in Class 2 (compared to Class 3) by 0.27 unit (relative probability of 21%) (95% confidence interval for *or*: 0.07–0.99).

#### Comparing Class 4 to Class 3

We found no significant differences between Class 3 and Class 4.

#### Comparing Class 5 to Class 3

Compared to no training, teachers receiving one training will decrease the relative likelihood of being in Class 5 (compared to Class 3) by 0.19 unit (relative probability of 16%) (95% confidence interval for *or*: 0.04–0.89). Compared to during the school year, teachers receiving training outside of the school year will increase the relative likelihood of being in Class 5 (compared to Class 3) by 2.60 unit (relative probability of 72%) (95% confidence interval for *or*: 1.37–4.94). Compared to costing under $100, teachers using products that cost $100 or more will increase the relative likelihood of being in Class 5 (compared to Class 3) by 4.17 unit (relative probability of 81%) (95% confidence interval for *or*: 1.54–11.31). Compared to schools in suburban locations, teachers from urban-located schools will decrease the relative likelihood of being in Class 5 (compared to Class 3) by 0.48 unit (relative probability of 33%) (95% confidence interval for *or*: 0.24–0.96). Compared to large districts, teachers at schools in medium-sized districts will decrease the relative likelihood of being in Class 5 (compared to Class 3) by 0.41 unit (relative probability of 29%) (95% confidence interval for *or*: 0.17–0.98).

## DISCUSSION

In this study we sought to understand elementary classroom teachers’ use of MI resources. Specifically, our research questions focused on (a) teachers’ perceptions regarding MI, including perceived benefits of MI, perceived barriers to using MI resources, satisfaction with MI resources they are using, perceived importance of different MI resource characteristics with respect to resource adoption, and MI preferences, (b) latent classes that exist based on teachers’ perceptions about MI, and (c) the extent to which teachers’ MI preferences, demographics and background, and school context characteristics predict class membership. We have organized our discussion in relation to each of these questions.

### Teachers’ Perceptions About MI

Descriptive statistics for the individual items revealed that most participants felt MI increases children’s PA and is beneficial to children’s learning and, particularly, on-task behavior. These results are promising since the teachers apparently did not see a conflict between using MI and promoting students’ academic performance, even though the results of previous studies showed that teachers felt the pressures involved with academic testing made incorporating PA opportunities for children a challenge (Michael et al., 2019). Participants in this study also agreed that MI makes learning more enjoyable for students and enhances the school’s image in the eyes of the school district. Further, participants felt that using MI had advantages in terms of its cost, convenience, and efficiency as a way to promote PA. As demonstrated in diffusion of innovations theory and research (Rogers, 2003), perceived relative advantage is a key predictor of the rate of innovation adoption. Webster et al. (2013) found a positive association between perceived relative advantage and self-reported use of MI in a study of elementary classroom teachers in South Carolina. Therefore, participants in the present study who more strongly agreed that MI is advantageous could also be more likely to adopt additional MI resources in the future. We also found a propensity for participants to feel that using MI gives them more control over promoting PA. As perceived behavioral control is an important determinant of intentionality within the theory of planned behavior (Ajzen, 1991), classroom teachers who feel that MI increases their control over promoting PA may be more likely to continue using MI in the future.

The response frequencies for the items assessing perceived barriers to using MI resources provided little clarity about any factors that are a particular concern for most teachers. In prior research, lack of time to implement MI was a predominant barrier for classroom teachers (Webster et al., 2019). Based on the results of the present study, however, we suggest that equal attention be given to all possible barriers (e.g., lack of time, cost of MI resources, lack of knowledge about MI) in professional development efforts, teacher education programs, or interventions. A large proportion of teachers selected the midpoint on the response scale for each item. Since we did not use anchors for the middle scale points, a rating of “3” could either suggest that the participants felt the factors listed on the questionnaire present a moderate level of challenge or that the participants were neutral in their feelings about the items. In spite of the mixed results, it is encouraging that there were no factors that emerged as a major barrier for the majority of participants.

Responses on items pertaining to different MI resource characteristics indicated that participants felt curriculum integration, time required for training, the types of training materials provided, and the timing of the training were most important to informing their decision to adopt a resource. Additionally, responses on items assessing MI preferences provided further details that would be helpful to those who develop MI resources and trainings. The options that participants agreed upon the most were for MI resources that (a) include a guidebook/training manual, (b) can be used within the classroom setting, (c) require no training or a training that lasts no longer than 90 min, and (c) can be implemented in a 6–20 min timeframe.

Exploratory and confirmatory factor analyses of the survey items assessing perceived benefits, perceived barriers to using MI resources, satisfaction with MI resources, and perceived importance of different MI resource characteristics supported a single-factor solution for benefits, barriers, and satisfaction, respectively, and a three-factor solution for resource characteristics (Curriculum Integration, Training Logistics, and Feasibility). For the present study, we used these factors in a person-centered analysis of the teachers’ MI perceptions. However, as we established these factors with a national sample, the factors may have further application in future research. For instance, researchers could also examine these factors using variable-centered analyses or person-specific analyses. According to Howard and Hoffman (2018), variable-centered approaches in research are used “to explain relationships between variables,” whereas person-centered approaches are used to “identify the dynamics of emergent subpopulations in a sample based on a chosen set of variables” (p. 848). Person-specific approaches take person-centered perspectives a step further by “[investigating] effects that may be idiosyncratic to specific subjects” (p. 851).

### Teacher Profiles

LPA results indicated there were five latent classes of teachers, based on factor scores for Benefits, Barriers, Satisfaction, Curriculum Integration, Training Logistics, and Feasibility. We viewed Latent Class 3 as the most desirable class when considering its members’ survey responses. Class 3 teachers were not extreme in their beliefs about MI; their rankings were neither highest nor lowest for any variable compared to teachers’ rankings in other classes, which may suggest an overall greater propensity for Class 3 teachers to be open-minded and adaptable to different MI approaches. Further, Class 3 teachers uniquely saw Curriculum Integration as relatively important compared to Training Logistics and Feasibility. These rankings seem to reflect a realistic appraisal of what features matter most in relation to using MI resources. Given that classroom teachers often follow very busy schedules with limited time to address additional initiatives (Cothran et al., 2010), it makes sense for teachers to place high importance on the ability of MI resources to align with the academic curriculum. Also, lower rankings on the importance of Training Logistics (amount of time required for training, cost of materials, and materials available) and Feasibility (delivery location, cost, and materials available) demonstrate a flexible attitude about the requirements needed to learn and implement MI, although Class 3 teachers’ rankings for these variables still reflected an appreciation that issues of logistics and feasibility must be given some priority.

### Predicting Class Membership

In the regression analyses, we used Class 3 as a referent group to examine the likelihood of teachers staying in or leaving other classes, based on teachers’ MI preferences, demographics and background, and school context characteristics. Since we viewed Class 3 as the most desirable profile, we interpreted results indicating a significant likelihood of leaving other groups in reference to Class 3 as favorable. We found that participants were significantly more likely to leave classes in which members prioritized Training Logistics and Feasibility when the teachers had at least one training for MI, taught in urban schools, taught in medium-sized school districts, were from minority racial/ethnic groups, or had an associate’s degree or some college education but had not attained a bachelor’s degree. Class membership was also more desirable when teachers preferred being given hard copy materials as part of MI training, preferred MI training that occurred during the school year, preferred less costly MI resources, and preferred using MI as part of an opening activity (i.e., start of school day) or as a reward for good student behavior. Overall, these results support previous research (Webster, 2011; Martin and Murtagh, 2017) in highlighting the importance of MI training in changing teachers’ beliefs about MI.

The lack of significant differences between Class 3 and Class 4 may be due to the fact that these classes were opposed to one another statistically. In particular, Class 3 teachers valued the importance of integrating MI resources with the academic curriculum, whereas Class 4 teachers did not. These classes also differed notably on several other variables. Class 4 teachers preferred implementation inside the classroom (compared to outside), while Class 3 teachers preferred external support training (compared to in person). Class 3 teachers were also more likely to be female and more likely to be less experienced (<5 years). Thus, it makes sense that the probability was low that Class 4 teachers would leave their class in reference to Class 3.

### Study Strengths and Limitations

This study is one of the first in which researchers have surveyed a national sample of elementary classroom teachers about MI. As with all survey research, this study is limited by the potential for response bias, inaccuracies associated with self-report data, and the inability to determine cause and effect due to the cross-sectional nature of the design. Another limitation is that the monetary incentive we offered to participants to complete the survey might have influenced participants’ responses. Nevertheless, this study is novel in that we assessed and compared different MI resource characteristics and also used a person-centered approach to identify distinct groups of teachers who value different MI-related characteristics. Further research is needed to explore the intrinsic value of MI for teachers, given MI’s education- and public health-related benefits. Qualitative studies would be beneficial in more deeply exploring the findings of the present investigation.

## CONCLUSIONS

Based on the findings from this study, we can glean new insights into why some MI resources may be more appropriate for teacher professional development, preservice teacher preparation, intervention programming, and future research aimed at increasing MI implementation. Considering the results holistically, we suggest that teachers may be more likely to seek and use MI resources that link with academic curricula. Thus, teacher education programs should primarily focus on helping teacher candidates learn to use MI resources that support the integration of PA with academic instruction and learning experiences. Further, we recommend that trainings, whether in the context of teachers’ continuing education/professional learning or intervention research, (a) be scheduled, when possible, in person and prior to or at the beginning of the school year, (b) include hard copy materials, and (c) focus on cost-effective strategies that can be used at the start of the school day and for incentivizing good student conduct. Teachers with less experience and who work at schools that may have fewer resources might be especially receptive to incorporating PA into academics and participating in different kinds of MI trainings in order to capitalize on all available methods for improving student outcomes. Researchers in future studies should investigate the effects of different types of MI training and MI resource characteristics on the beliefs of teachers who work in different school contexts and belong to the different profiles (i.e., latent classes). Finally, given the current public health crisis related to the COVID-19 pandemic, both researchers and practitioners should seek to identify and leverage MI resources that best promote MI as an embodied and routine practice for teachers, their students, and students’ parents in contexts that demand virtual learning and social distancing.

## Figures and Tables

**TABLE 1 |:** Characteristics of participant schools and teachers.

School characteristics	*n*	%
**SAMPLE CHARACTERISTICS**		
**Geographic area**		
Urban	147	35
Suburban	203	48
Rural	70	17
**District size (%)**		
Small district (< 10 schools)	221.0	53
Medium district (10–25 schools)	133.0	32
Large district (> 25 schools)	66.0	16
**Type of school**		
Public	326.0	78
Private	94.0	22
**Majority of students qualify for**		
Free lunch	221.0	53
Reduced price lunch	118.0	28
No free/reduced cost lunch	81.0	19
**PARTICIPANT TEACHER CHARACTERISTICS**		
Female	331	79
**Years of experience**		
≤ 5	163	39
6–10	130	31
11–15	88	21
≥20	39	9
**Race/ethnicity**		
African American	64	15
Asian	12	3
White	285	68
Hispanic	50	12
Other	9	2
**Teacher education**		
Some college	16	4
4-year bachelor’s degree	282	67
Graduate degree	122	31

**TABLE 2 |:** Teachers’ response frequencies for each survey item.

PERCEIVED BENEFITS OF MOVEMENT INTEGRATION							
Root	Stem	strongly disagree	Disagree	Slightly disagree	Slightly agree	Agree	Strongly agree
As you think about MI as a possible part of your classroom routines, how strongly do you agree or disagree?	Compared to typical classroom routines, implementing MI is more costly	84	61	47	53	34	68
	Implementing MI enhances learning for my students	19	16	16	42	109	145
	Implementing MI keeps my students on task better than other activities	19	15	15	45	113	140
	Implementing MI makes learning more fun for my students	25	13	15	35	92	167
	Offering MI enhances how our school is viewed by other schools in our district.	41	13	21	49	104	119
	Providing MI enhances my performance as a teacher	34	12	20	45	102	134
	Implementing MI is a more convenient way to promote physical activity	27	13	21	35	107	144
	Offering MI allows me to promote physical activity more efficiently	28	13	15	39	90	162
	Using MI gives me more control over promoting physical activity	25	16	22	43	115	126
	Implementing MI increases students’ participation in physical activity	31	15	13	33	103	152
	Implementing MI enhances students’ knowledge about the importance of physical activity	30	10	20	29	96	162

PERCEIVED BARRIERS TO MOVEMENT INTEGRATION RESOURCE USE						
Root	Stem	Not a barrier at all 1	2	3	4	Major barrier 5
How would you rate each of the following barriers as challenges to using MI products in your classroom routines?	Limited planning time	75	48	95	49	80
	Limited instructional time	65	54	91	84	53
	Cost	51	49	89	83	75
	Lack of formal MI training	45	61	92	96	53
	Lack of information/knowledge about MI	54	58	87	84	64
	Not my responsibility	139	50	64	38	56

SATISFACTION WITH MOVEMENT INTEGRATION RESOURCES				
	Extremely dissatisfied	Slightly dissatisfied	Slightly satisfied	Extremely satisfied
How satisfied are you with the current MI approach/products you are using?	0	13	139	195
	Extremely unlikely	Somewhat unlikely	Somewhat likely	Extremely likely
How likely are you to recommend the product you are using to other classroom teachers?	2	10	113	222

PERCEIVED IMPORTANCE OF DIFFERENT MOVEMENT INTEGRATION RESOURCE CHARACTERISTICS									
		Importance							
Root	Stem	Most 1	2	3	4	5	6	7	Least 8
There are many elements of a comprehensive MI product. Please rank the following product characteristics on the importance to you and your classroom routines.	Amount of time required for teacher training	108	54	51	30	26	20	22	31
	The type of training materials provided (online vs. hard copy, in person vs. virtual, etc).	68	70	40	41	31	40	29	21
	Timing of training (before or after the school day, beginning, or end of the year, etc).	58	58	49	43	44	34	32	20
	The actual integration into the curriculum (aligns with other academics, use, delivery).	87	75	43	49	34	23	17	12
	Delivery location (classroom, lab, outdoors, etc).	44	51	30	42	63	36	34	38
	Length of time for the activity.	54	58	40	32	38	66	33	20
	Cost of the product.	81	43	29	26	18	36	73	33
	Specific materials available/included in the product.	29	39	30	26	34	32	45	102

MOVEMENT INTEGRATION RESOURCE PREFERENC						
How much did you pay (or your school pay) for the MI product(s) you are currently using or have used most recently?	**Free**	**< $50**	**$50–$99**	**$100–$199**	**$200–$299**	**$300–$399**
	57	47	58	53	37	15
Based on your needs and teaching plan, what is the optimal duration of an MI product training for you to get comfortable with implementing the product?	**No training Is required**	**60 min**	**90 min**	**Half day**	**Full day**	**Multiple days**
	50	134	48	31	54	30
With many of these products a guidebook may be provided to support you as you become familiar with the new MI product. Which format do you prefer for this guidebook?	**None**	**Hard copy/Paper**		**Digital manual**		**Virtual/Online (a link)**
	24	123		112		88
If you were to receive professional training and development support for implementing an MI product, which form of support would you prefer?	**In person**	**Webinar**		**Videos**		**External support (e.g., phone or web based technical support available)**
	156	74		85		32
In thinking about your school year, when would be the best timing to receive MI training and support?—Selected choice	**Prior to the start of the school year**	**Beginning of the school year**	**End of school year**	**Following the end of the school year**	**Mid School year**	**Communications throughout the year**
	185	93	27	19	10	12
As you think about the school day, if you were to receive ongoing communications about training and support for these types of products what time of day is best for you to receive this type of support?	**Before the school day starts**		**During the school day**		**After the school day**	
	138		4		205	
Please select your preferred approach to integrating these products into your classroom routines	**Integrated within academic lessons/content**			**As a break from/transition between academics**		
	173			174		
What is your preferred method for implementing MI products into your classroom routines?	**Technology Led (e.g., online video or game)**	**Teacher Led (e.g., you lead students through an activity)**	**Student Led (e.g., students lead other students through an activity)**		**Equipment Led (e.g., standing desks, chairs that allow children to wobble/move, etc.)**	
	108	169	54		16	
Where do you most often use MI?	**General Classroom**	**Activity Lab (e.g., room with special equipment to deliver integrated content)**	**Outdoors**	**Gymnasium**	**Other shared facility (e.g., cafeteria, auditorium, etc…)**	
	240	30	57	19	1	
How long is your typical or average MI occasion?	**<5min**	**6–10 min**	**11–15 min**	**16–20 min**	**21–30 min**	**Over 30 min**
	33	88	76	68	50	32

**TABLE 3 |:** Factor loadings from the confirmatory factor analyses.

	Item	Standardized factor loading	Residual variance
**PERCEIVED BENEFITS OF MI RESOURCES**			
	On task	0.865	0.252
	Learning fun	0.843	0.29
	Visibility	0.853	0.273
	Teach performance	0.807	0.35
	Convenient PA	0.858	0.264
	Promote PA efficient	0.884	0.218
	Promote PA control	0.869	0.245
	Increase PA	0.86	0.26
	participation		
	Increase PA knowledge	0.855	0.268
**PERCEIVED BARRIERS TO MI RESOURCES**			
	Planning time	0.699	0.512
	Instruction time	0.711	0.494
	Cost	0.623	0.612
	Training	0.711	0.495
	Knowledge	0.761	0.422
	Responsibility	0.421	0.823
**CHARACTERISTICS OF MI RESOURCES AND TEACHER SATISFACTION**			
Satisfaction	Satisfaction	0.885	0.217
	Likeliness to recommend	0.644	0.585
Training logistics	Training	0.605	0.634
	Training materials	0.545	0.703
	Training timing	0.659	0.566
Feasibility	Delivery location	0.514	0.736
	Cost	0.671	0.55
	Available materials	0.74	0.453

**TABLE 4 |:** Fit values for the different class solutions.

	2-Class	3-Class	4-Class	5-Class
Loglikelihood	−3917.64	−3850.67	−3796.30	−3759.29
AIC	7879.28	7761.34	7668.60	7610.57
BIC	7963.97	7876.82	7814.87	7787.64
Entropy	0.93	0.95	0.90	0.92
VLMR p-value	0.0009	0.0001	0.5311	0.0106
LMR p-value	0.0010	0.0002	0.5365	0.0112
Class 1	14%	14%	7%	16%
Class 2	86%	84%	62%	13%
Class 3		2%	29%	20%
Class 4			2%	14%
Class 5				36%

**TABLE 5 |:** Latent profile conditional response means and overall sample means.

Variable/Factor	Sample Mean (*n* = 347) (SD)	Class 1 (*n* = 56) (SD)	Class 2 *(n* = 46) (SD)	Class 3 (*n* = 71) (SD)	Class 4 (*n* = 50) (SD)	Class 5 (*n* = 124) (SD)
Benefits of movement integration	4.98 (1.10)	4.01 (1.01)	5.36 (1.01)	5.18 (1.01)	5.19 (1.01)	5.07 (1.01)
Barriers to movement integration resource use	3.01 (0.98)	2.95 (0.95)	2.61 (0.95)	3.00 (0.95)	2.83 (0.95)	3.27 (0.95)
Satisfaction	3.56 (0.51)	3.44 (0.50)	3.50 (0.50)	3.49 (0.50)	3.61 (0.50)	3.67 (0.50)
Training Logistics	3.62 (1.71)	1.43 (0.61)	6.50 (0.61)	4.72 (0.61)	4.25 (0.61)	2.65 (0.61)
Feasibility	4.55 (1.89)	1.65 (0.71)	3.11 (1.89)	4.82 (1.89)	3.94 (1.89)	6.50 (1.89)
Curriculum Integration	3.18 (1.99)	1.67 (1.34)	2.63 (1.34)	2.04 (1.34)	6.43 (1.34)	3.42 (1.34)

**TABLE 6 |:** Odds ratios and p-values for the logistic regression model.

Variable	Categories	Class 1		Class 2		Class 4		Class 5	
		odds ratio	*P*	Odds ratio	*P*	Odds ratio	*P*	Odds ratio	*P*
Preferred approach integrated	Integrated within lesson	1.88	0.13	0.56	0.17	0.73	0.42	1.23	0.53
	Transition between academics	ref	ref	ref	ref	ref	ref	ref	ref
Preferred approach opening activity	Daily opening activity	2.65	0.02	0.56	0.23	1.92	0.11	1.30	0.46
	Throughout the day	ref	ref	ref	ref	ref	ref	ref	ref
Preferred approach reward	Reward for good behavior	3.19	0.01	1.97	0.14	1.55	0.31	1.91	0.08
	Used as part of curriculum	ref	ref	ref	ref	ref	ref	ref	ref
Preferred implementation method	Student	0.58	0.39	1.57	0.45	1.18	0.77	0.50	0.17
	Teacher	1.03	0.94	1.18	0.73	1.04	0.94	0.72	0.37
	Technology/Equipment	ref	ref	ref	ref	ref	ref	ref	ref
Preferred implementation location	Classroom	0.30	0.00	1.31	0.58	1.10	0.83	1.19	0.63
	Outside classroom	ref	ref	ref	ref	ref	ref	ref	ref
Preferred implementation length	10 min or less	1.09	0.91	0.85	0.84	0.56	0.45	0.39	0.14
	11–30 min	1.00	1.00	0.46	0.35	0.96	0.96	1.05	0.94
	Over 30 min	ref	ref	ref	ref	ref	ref	ref	ref
Current resource cost	Free	4.88	0.03	2.61	0.08	1.21	0.73	1.86	0.29
	Under $100	7.46	0.00	3.21	0.03	1.18	0.74	4.17	0.01
	$100 or more	3.20	0.08	0.39	0.21	0.46	0.15	5.33	0.00
	Unsure	ref	ref	ref	ref	ref	ref	ref	ref
Training length	None	0.15	0.02	2.11	0.44	1.14	0.88	0.19	0.04
	One	0.23	0.03	1.00	1.00	0.85	0.84	0.62	0.44
	Multiple	ref	ref	ref	ref	ref	ref	ref	ref
Training delivery method	Digital manual	1.13	0.84	0.47	0.17	0.92	0.87	1.22	0.62
	Hard copy	4.20	0.01	1.44	0.45	1.59	0.34	0.95	0.91
	None	12.98	0.01	1.98	0.48	1.47	0.72	3.41	0.17
	Online	ref	ref	ref	ref	ref	ref	ref	ref
Training support	External support	0.72	0.70	0.63	0.54	1.18	0.82	0.83	0.75
	In person	1.75	0.29	0.89	0.82	1.86	0.24	1.40	0.42
	videos	1.35	0.60	0.86	0.78	1.04	0.95	0.59	0.24
	Webinar								
Training time of year	During school year	2.93	0.01	1.12	0.80	0.98	0.95	2.60	0.00
	Not during school year	ref	ref	ref	ref	ref	ref	ref	ref
School location	Rural	0.50	0.28	3.63	0.06	1.96	0.30	0.54	0.27
	Suburban	0.36	0.02	1.48	0.47	1.17	0.74	0.48	0.04
	Urban	ref	ref	ref	ref	ref	ref	ref	ref
School district size	Large	0.24	0.02	0.27	0.05	0.46	0.17	0.41	0.05
	Medium	0.78	0.58	0.50	0.14	0.48	0.12	1.11	0.77
	Small	ref	ref	ref	ref	ref	ref	ref	ref
School type	Private	0.47	0.15	0.69	0.47	0.70	0.48	1.08	0.85
	Public	ref	ref	ref	ref	ref	ref	ref	ref
School size	250 or less	3.04	0.19	2.84	0.38	2.79	0.28	1.39	0.65
	251–500	0.42	0.32	1.75	0.64	1.03	0.97	0.92	0.90
	501–1,000	0.75	0.74	2.30	0.48	0.96	0.96	1.48	0.57
	Over 1,000 students	ref	ref	ref	ref	ref	ref	ref	ref
Free/reduced lunch	Free lunch	0.76	0.62	0.64	0.38	1.77	0.33	1.39	0.46
	Reduced lunch	0.75	0.63	0.37	0.10	1.77	0.37	1.09	0.86
	None	ref	ref	ref	ref	ref	ref	ref	ref
Teacher gender	Female	1.09	0.85	1.29	0.63	1.15	0.77	0.53	0.08
	Male	ref	ref	ref	ref	ref	ref	ref	ref
Teacher years experience	5 or less	1.68	0.27	0.62	0.31	0.55	0.22	0.89	0.76
	6–10 years	1.68	0.37	1.23	0.71	1.99	0.18	1.29	0.57
	11–20 years	0.59	0.49	0.91	0.88	0.81	0.74	0.42	0.11
	20 or more	ref	ref	ref	ref	ref	ref	ref	ref
Teacher ethnicity	Non-hispanic white	0.44	0.04	1.36	0.49	1.17	0.73	0.90	0.75
	Minority	ref	ref	ref	ref	ref	ref	ref	ref
Teacher education	Non-bachelors	2.83	0.05	1.46	0.55	2.25	0.16	1.46	0.43
	Bachelors	0.43	0.07	1.32	0.54	1.04	0.93	0.63	0.20
	Graduate	ref	ref	ref	ref	ref	ref	ref	ref

Class 3 was used as the referent group.

## Data Availability

The datasets generated for this study are available on request to the corresponding author.
